# Genetic Variability and Evidence of a New Subgroup in Watermelon Mosaic Virus Isolates

**DOI:** 10.3390/pathogens10101245

**Published:** 2021-09-26

**Authors:** Osama A. Abdalla, Akhtar Ali

**Affiliations:** 1 Department of Biological Science, The University of Tulsa, Tulsa, OK 74104, USA; osama-mohammad@utulsa.edu; 2 Department of Plant Pathology, Faculty of Agriculture, Assiut University, Assiut 71526, Egypt

**Keywords:** negative selection pressure, recombination, coat protein

## Abstract

Watermelon mosaic virus (WMV) is one of the important *Potyviruses* that infect cucurbits worldwide. To better understand the population structure of WMV in the United States (U.S.), 57 isolates were collected from cucurbit fields located in nine southern states. The complete coat protein gene of all WMV isolates was cloned, sequenced and compared with 89 reported WMV isolates. The nucleotide and amino acid sequence identities among the U.S. WMV isolates ranged from 88.9 to 99.7% and from 91.5 to 100%, respectively. Phylogenetic analysis revealed that all the U.S. WMV isolates irrespective of their geographic origin or hosts belonged to Group 3. However, the fifty-seven isolates made three clusters in G3, where two clusters were similar to previously reported subgroups EM1 and EM2, and the third cluster, containing nine WMV isolates, formed a distinct subgroup named EM5 in this study. The ratio of non-synonymous to synonymous nucleotide substitution was low indicating the occurrence of negative purifying selection in the CP gene of WMV. Phylogenetic analysis of selected 37 complete genome sequences of WMV isolates also supported the above major grouping. Recombination analysis in the CP genes confirmed various recombinant events, indicating that purifying selection and recombination are the two dominant forces for the evolution of WMV isolates in the U.S.

## 1. Introduction

Cucurbits are important cash crops in the southern United States (U.S.). Unfortunately, more than 96 viruses [1] infect cucurbit crops naturally, and watermelon mosaic virus (WMV) is one of the important viruses that has a greater impact on the quality and quantity of cucurbits worldwide. WMV is a member of genus *Potyvirus*, family *Potyviridae*, which has flexuous rod-shaped particles of approximately 750 nm in length [2,3] and is transmitted by various aphids in a non-persistent manner [4,5]. Like other *Potyviruses*, the genome of WMV is a single-stranded positive-sense RNA and contains one large open reading frame, which translates into one large polypeptide of 3217 amino acids, including nine putative cleavage sites that produce ten different proteins [6,7]. WMV is most related to soybean mosaic virus (SMV) [8], suggesting that WMV has evolved as a result of recombination between SMV and bean common mosaic virus (BCMV) [9]. 

WMV is economically important and can infect more than 170 species, belonging to 27 different families, causing severe losses in important horticulture crops, especially cucurbits [9,10]. In cucurbits, WMV induces a variety of symptoms, according to the isolates and host cultivar that include: mosaic, vein banding, severe leaf deformation and filiformy, and some isolates induce discoloration and deformation, especially in squash fruits [4,5,11]. WMV is considered among the most important aphid-borne viruses infecting cucurbits worldwide, especially in temperate and Mediterranean climates [12,13,14].

In the U.S., WMV was first reported in 1965 [15] from southern Texas and is one of the main viruses infecting cucurbits in Texas [16], California [3], New Jersey [17], Illinois [18], Oklahoma [19] and other southern states [11]. Although WMV has been reported in the U.S. for more than 50 years, very little information is available about the genetic diversity of WMV in the U.S. 

Most studies about WMV variability came from Europe, where emergent strains of WMV caused severe symptoms in cucurbits [20]. WMV isolates were classified into three molecular groups (G1-G3) based on the 3–5 amino acid (aa) motif deduced from the 218 nucleotides (nt) at the coat protein (CP) N-terminal region [21] which were recognized by monoclonal antibodies made against these specific amino acids. Group 1 (G1) (also known as classical (CL) isolates) and Group 2 (G2) isolates have KEA and KET aa motifs, respectively, at position 3–5 in the N-terminal part of the CP, while Group 3 (G3) isolates (also named emerging (EM) isolates) have a KEKET aa motif at position 3–7 (with the insertion of two aa) in the N-terminal part of the CP [21]. G3 (EM) isolates have been further divided into four subgroups (E1–E4) [20,22].

One study [23] reported the genetic diversity of 36 WMV isolates based on the CP N-terminal region from Slovakia and Iran, and showed that WMV isolates from Slovakia belong to G1 and G2 groups, while isolates from Iran belong to the G2 group only. Recently, genetic diversity of 56 WMV isolates from China [24], using complete CP gene nt sequences, showed that all Chinese WMV isolates belong to G3. In the U.S., the only available data about WMV isolates came from Quemada et al. [25], who reported that the deduced amino acid sequence of the CP gene of the WMV isolate (USA90-Accession no. D13913) shared 94.7% similarity with other WMV worldwide isolates and 82.6% with soybean mosaic virus (SMV). Desbiez et al. [21] used two U.S. WMV isolates (USA90 and USA92, Accession no. DQ845032) in comparison, and they were placed in G2 and G3, respectively.

The aim of this present study is to provide more information about the genetic diversity of WMV isolates collected from cucurbit fields in nine southern states in the USA and determine their relationship with the worldwide WMV isolates, based on the CP gene. This information will be helpful to determine the evolutionary relationships of the U.S. WMV isolates, and to increase our understanding of how this virus evolved over time. Information about genetic diversity is essential for the control of plant viruses, using transgenic plants, and could reduce the impact of a virus on the quality and quantity of cucurbits. 

## 2. Results

### 2.1. Genetic Diversity of WMV in the U.S.

The genetic diversity of the U.S. population of WMV was analyzed by comparing the nucleotide sequences of the CP gene. Sequencing of the CP gene showed that all the U.S. WMV isolates have 849 nt, which could be translated into 283 aa. No insertion or deletion was found but substitution was common among the isolates. Nucleotide sequence identities among the WMV isolates ranged from 88.9 to 99.7% at nt and from 91.5 to 100% at aa level, respectively. The DAG motif, which is responsible for aphid transmission in *Potyviruses* [26] was found in all the U.S. isolates. 

Initially, phylogenetic trees were constructed based on the complete CP sequences of 57 WMV isolates according to the location (states) from where they were originally collected (Table 1). Phylogenetic trees of WMV isolates from AR, FL, MS, OK and TX states comprised two main groups (data not shown). The sequence identities among the WMV isolates according to the location ranged from 91.2 to 98.3% at nt and from 93.6 to 97.5% at aa (AR), 99.5–100% nt and 99.2–100% aa (FL), 91.6–99.2% nt and 93.9–98.9% aa (MS), 88.4–100% nt and 88.3–100% (OK), 89.5–100% nt and 91.8–100% aa, respectively. The number of isolates from other states (AL, GA, LA, and KY) was low and these were grouped together where the sequence identities among the WMV isolates ranged from 90.4 to 99.5% nt and from 92.2 to 99.5% aa, respectively. 

Similarly, average evolutionary divergence within and between the WMV isolates collected from the respective states was calculated using MEGA7. The divergence in the complete CP nt sequences was highest within isolates from OK (0.0631 ± 0.0057), followed by isolates from other states (AL, GA, LA and KY) (0.0624 ± 0.0066), AR isolates (0.0572 ± 0.0049), TX isolates (0.0559 ± 0.0049), MS isolates (0.0487 ± 0.0056), and FL isolates (0.0057 ± 0.0018). The divergence among the 57 WMV isolates from nine different states varied from 0.05 to 0.07. 

### 2.2. Phylogenetic Analysis of the CP Gene

**Figure 1:**
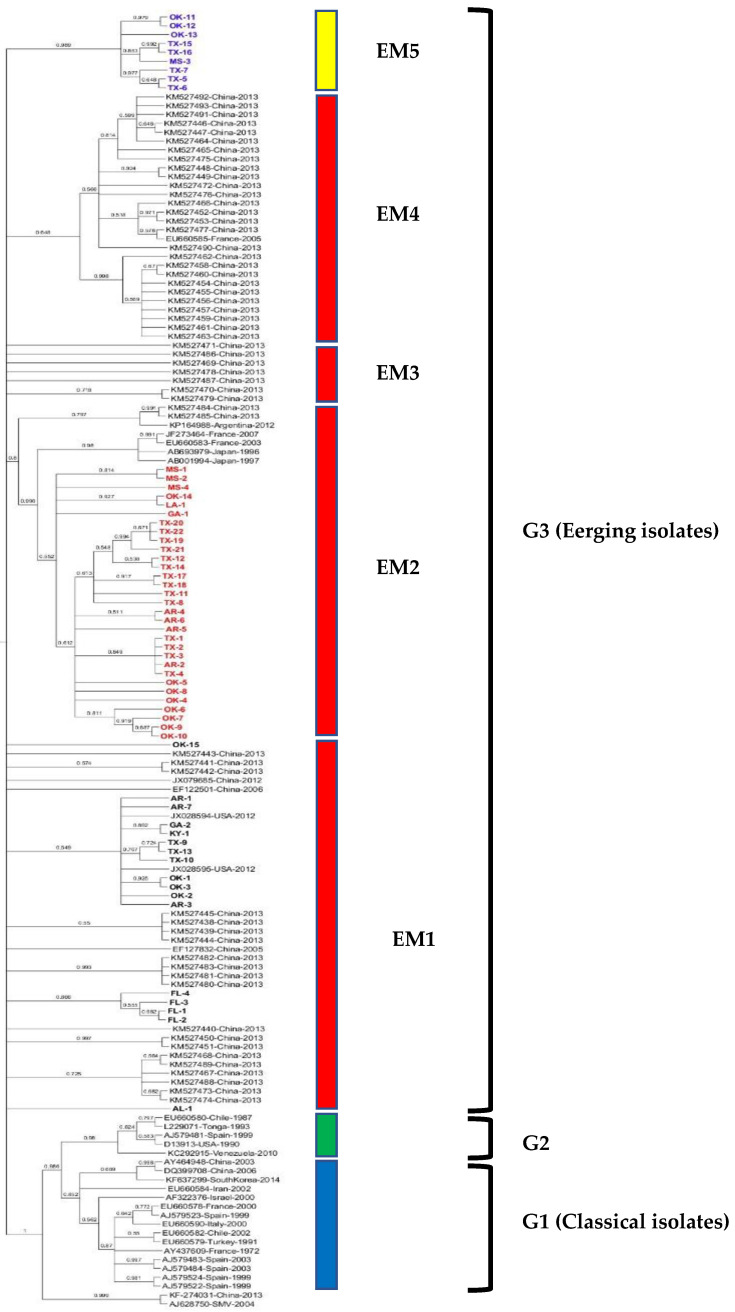
Maximum likelihood (ML) tree showing the phylogenetic relationships based on the nucleotide sequences of complete coat protein (CP) genes among 57 U.S. WMV isolates obtained in this study and 89 published WMV isolates reported worldwide previously (available from GenBank). In the ML trees, the names of U.S. WMV isolates obtained in this study are without accession number and denoted by the abbreviation of various states and number of isolates. Detailed information for the U.S. WMV isolates is listed in Table 1, while sequences of the 89 isolates from other countries that were downloaded from the GenBank are listed in Table S1. ML trees were generated using MEGA7 with the Tamura-Nei model (TN93+G+I) model. Bootstrap values (1000 replicates) greater than 50% are indicated at the tree nodes. soybean mosaic virus (SMV) was used as the outgroup.

The maximum likelihood tree was constructed from the complete CP gene nt sequence of all 57 WMV isolates along with 89 WMV isolates (Table S1) reported worldwide. The phylogenetic tree shows that all 57 WMV isolates clustered with G3 (EM isolates) (Figure 1) but in various subgroups within G3. For example, subgroup EM1 included 17 WMV isolates (1 each from AL, GA and KY, 3 each from AR, and TX, and 4 each from FL and OK). Similarly, subgroup EM2 contained 31 WMV isolates (1 each from LA, and GA, 3 from MS, 4 from AR, 8 from OK, and 14 isolates from TX). Subgroup EM5 (named in this study) contained nine WMV isolates (one from MS, three from OK, and five from TX) and formed a separate distinct cluster from the published WMV isolates used in the phylogenetic analysis (Figure 1).

Intragroup variability in the complete CP nt sequence in subgroup EM5 was higher (0.0337 ± 0.0039) than subgroups EM1 (0.0218 ± 0.0023) and EM2 (0.0218 ± 0.0024). Similarly, intergroup variability was higher between subgroups EM5 and EM1 (0.09), EM5 and EM2 (0.09) than subgroups EM1 and EM2 (0.08).

### 2.3. Amino Acid Sequence Comparison

**Figure 2:**
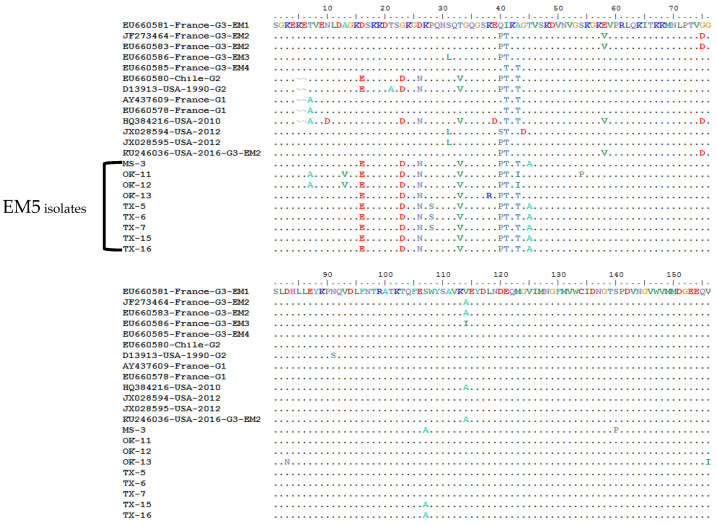

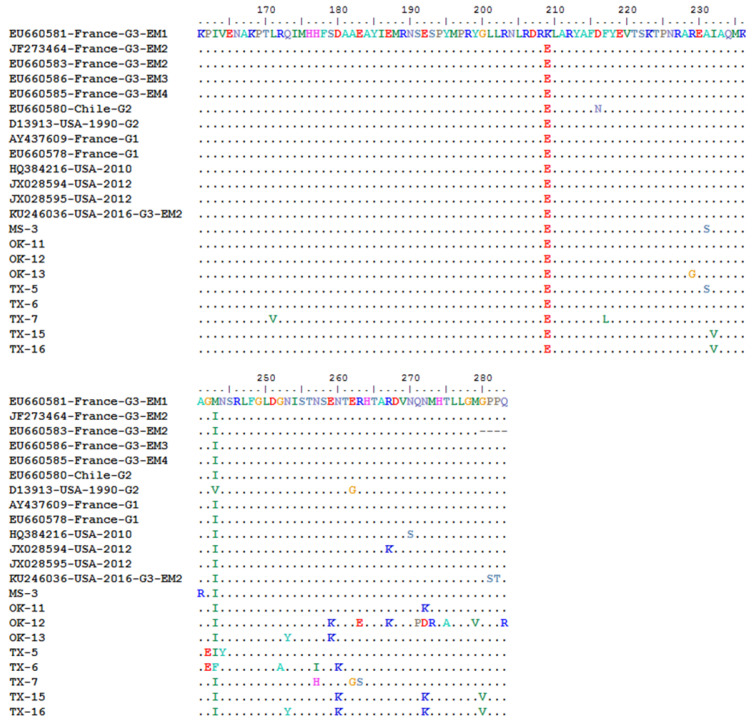
Multiple sequence alignment of the complete coat protein gene amino acids (283 aa) of EM5 subgroup watermelon mosaic virus (WMV) isolates from the U.S. and their comparison with the representative WMV isolates from G1, G2, and G3 groups reported from other countries of the world. The three WMV isolates reported previously from the U.S. are also included for comparison. In the top panel, the horizontal boxes show the specific conserved amino acid motifs in the N-terminal of the CP gene among the G1, G2, and G3 isolates. Vertical boxes show specific conserved amino acids that only exist in EM5 subgroups isolates.

In order to determine any subgroup’s specific amino acid pattern in the CP gene, the 283 aa sequences of the 57 WMV isolates were compared with the representative isolates that were previously classified into G1, G2 and G3 isolates (Figure 2). All G3 isolates carry a specific motif (KEKET) at position 3–7 in the N-terminal of CP gene, while G1 and G2 isolates have KET and KEA at the same positions (Figure 2). All of the 57 WMV isolates had the KEKET aa motif except for two isolates (OK-11 and OK-12), which were unique and have “A” at position 7 instead of “T” (KEKEA instead of KEKET) (Figure 2). In addition, they also had a unique aa: V at position 28, which did not exist in any WMV isolates (Figure 2). Both OK-11 and OK-12 WMV isolates clustered in the new subgroup EM5, which was identified in this work. 

Further analysis of the aa sequence comparison showed that some of the WMV isolates in subgroup EM5 had 10 unique and conserved aa: V, S, I, A, A, Y, K, K, K, and V at positions 13, 28, 43, 45, 107, 253, 259, 260, 272, and 280, respectively (Figure 2). Among these 10 unique aa, four aa (V, S, I and A) at positions 13, 28, 43, and 45 were located at the N-terminal of the CP gene while the remaining were located at the C-terminal of CP gene except one at positions 107 which was in the core region of CP gene. Apart from the specific aa of WMV isolates in subgroup EM5, there were four aa: E, D, N, and V, at positions 16, 23, 26, 33, which were common between EM5 and G2 isolates (Figure 2), while there was one aa (T) which was common between EM5, G1, and G2 isolates. 

**Figure 3:**
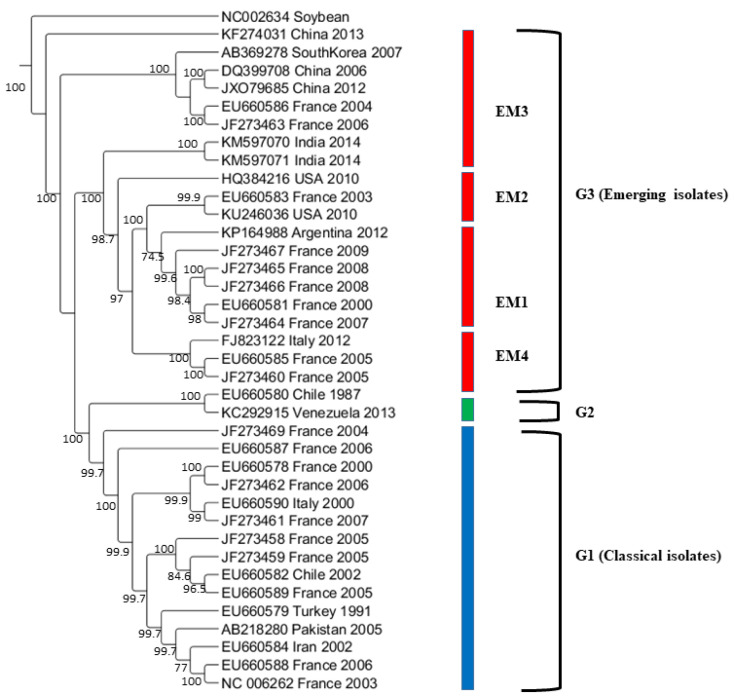
Maximum likelihood (ML) tree showing the phylogenetic relationships based on the nucleotide sequences of complete genome sequences of 37 WMV isolates containing 2 U.S WMV isolates and 35 other WMV isolates reported worldwide previously (available from GenBank). ML trees were generated using MEGA7 with the Tamura-Nei model (TN93+G+I) model. Bootstrap values (1000 replicates) greater than 50% are indicated at the tree nodes. soybean mosaic virus (SMV) was used as an outgroup.

The U.S. isolate from Texas (Accession no. KU246036) that was classified as G3-EM2 isolate by phylogenetic analysis (Figure 3) shared three aa (V, D and A) at positions 58, 75 and 114 with G3-EM2 isolates (Figure 2), which further confirmed that it is the G3-EM2 isolate. The other U.S. isolate (Accession no. HQ384216) collected from a weed and classified as a G1 isolate based on the presence of KEA at the N-terminal of CP gene (Figure 2) showed similar aa (V, D, and A) at positions 58, 75, and 114, just like G3-EM2 isolates which were similar as shown in the phylogenetic analysis (Figure 3) where they clustered with G3 isolates. 

### 2.4. Selection Pressure in the CP Gene 

The selection pressure on the CP gene of WMV isolates for the three subgroups (EM1, EM2 and EM5) was determined by calculating dN/dS (ω) ratio (Table 2). The dN/dS ratio for the three subgroups, EM1 isolates (0.219), EM2 isolates (0.3089) and EM5 isolates (0.2075), indicated that negative (or purifying) selection is occurring across WMV isolates in the CP gene. The strength of the negative selection was higher among the subgroup EM2 isolates as compared to subgroups EM1 and EM5 isolates, indicating that stronger constraints are operating in the CP genes of WMV isolates among the later two groups. Similarly, the dN/dS ratio in the CP gene was also estimated according to the origin of collection of WMV isolates. The dN/dS ratio was 0.0859 (AR isolates), 0.3170 (FL isolates), 0.0936 (MS isolates), 0.0940 (OK isolates), 0.0827 (TX isolates), and 0.0847 for WMV isolates (Table S2) collected from AL, GA, LA, and KY states, and also showed that negative selection occurred in WMV isolates collected from nine different states. However, the strength of negative selection was stronger in all WMV isolates except FL WMV isolates, which showed weaker negative selection compared with the isolates from other southern states. 

Selection pressure calculated at the level of individual codons by the three codon-based methods (SLAC, FEL, and REL) among the three subgroups mostly showed that a number of codons are under negative selection (Table 2). However, a total of 16 codons in EM2 and 3 codons in EM5 were under positive selection as detected by the REL algorithm only. The ENC indicates a positive correlation with the rate of synonymous substitution. For example, EM1 and EM5 showed stronger codon bias (ENC of 50) compared with the EM2 subgroup isolates (ENC 51), confirming a stronger purifying selection in these two group isolates. 

**Figure 4:**
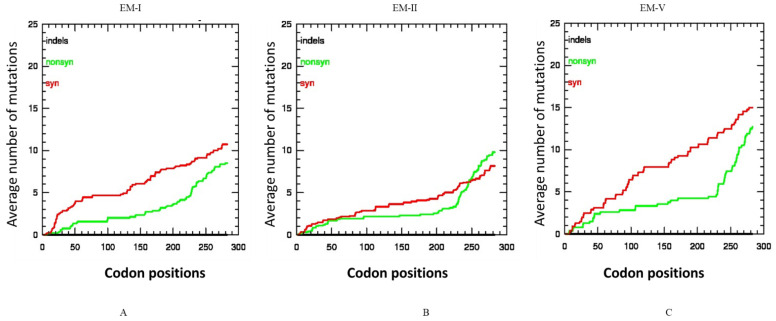
Average incidences of synonymous, and non-synonymous and indel mutations in the codons of the coat protein (CP) gene of watermelon mosaic virus isolates subgroups: (**A**) EM1 isolates, (**B**) EM2 isolates, (**C**) EM5 isolates. The *X*-axis represents the position of the codon while the *Y*-axis represents the average cumulative number of synonymous, non-synonymous mutations estimated at specific codon positions in the CP gene. No indel was present in the CP of any WMV isolates in the three subgroups.

The selective constraints in the CP gene of the three subgroup isolates were also determined by analyzing the distribution of synonymous and non-synonymous and indel mutations. In the EM2 subgroup the number of synonymous and non-synonymous mutations was not significantly different (Figure 4A), but in the case of EM1 and EM5 subgroups (Figure 4B,C), the synonymous mutations were higher than the non-synonymous mutations. No indel was present in any WMV isolates among the three subgroups.

### 2.5. Genetic Differentiation and Gene Flow Analysis of WMV Isolates

Genetic differentiation and gene flow analysis (Table 3) estimated from F statistics (*Fst*) indicated that there is frequent gene flow of WMV isolates among different states (*Fst <* 0.33) except between Arkansas and Florida (*Fst* = 0.439), Mississippi and Florida (*Fst* = 0.620), Oklahoma and Florida (*Fst* = 0.470), Texas and Florida (*Fst* = 0.569) and Florida and other states (*Fst* = 0.270). Similarly, the Nm value for all states pairs was >1 except different states vs. Florida (<1) indicating frequent gene flow among WMV isolates. The *p* values for all permutation-based tests Ks, Ks*, Z* and Snn were <0.01 for all WMV isolates from states pairs involving Florida, showing significant genetic differentiation (Table 3).

### 2.6. Co-Evolution Analysis of CP Gene Sequences

Analysis of BGM showed 16 co-evolving codon pairs with a Bayesian posterior probability of at least 50% in the CP gene of WMV isolates (Table S4). Most of the co-evolving sites were located on the C-terminal of the CP gene.

### 2.7. Phylogenetic Analysis of the Complete Genome WMV Isolates

The maximum likelihood tree was constructed from the complete genome sequences of 37 representatives WMV isolates (Figure 3). The phylogenetic tree shows that that the 37 isolates formed three major groups (G1, G2, and G3) as obtained from the CP gene sequences [21]. In G3, WMV isolates were further divided into four subgroups that included EM1, EM2, EM3, and EM4 (Figure 3) as described previously [20]. Our phylogenetic analysis showed that U.S. WMV isolates from Texas belonged to the EM2 subgroup within G3. Classification based on the complete genome sequences showed additional evidence that grouping obtained on the basis of CP gene sequences and complete genome sequences are somewhat similar. 

**Figure 5:**
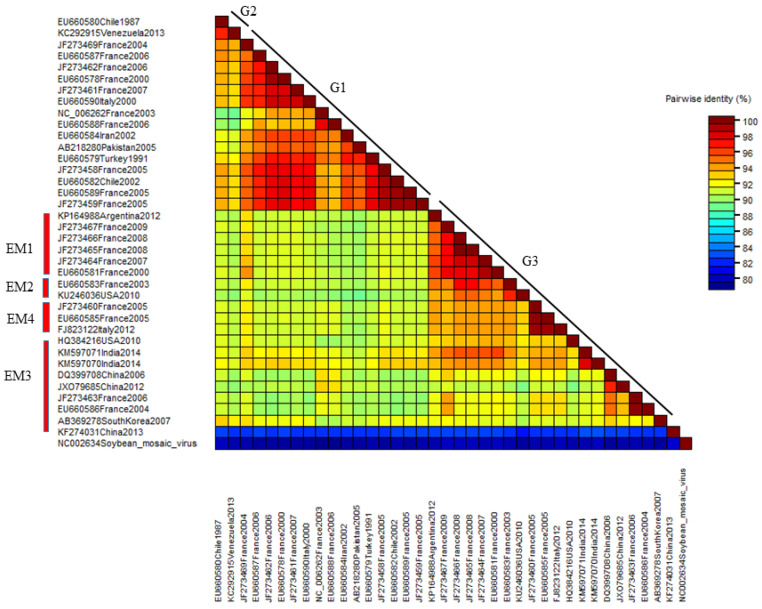
The distribution of pairwise identity score of the complete genome sequences of 37 WMV isolates as determined by the MUSCL multiple sequence alignment program in SDT software version 1.2. All 37 isolates have been shown in three molecular groups (G1, G2 and G3).

The nucleotide pairwise genetic identities between the complete genome sequences of 37 WMV isolates was calculated by SDT software (Figure 5). WMV isolates in G2 showed >98% pairwise identities followed by G1 isolates that ranged from 88 to 98%. G3 isolates showed the most diversity, and the lowest pairwise identity ranged from 82 to 98%.

### 2.8. Recombination Events within the CP Gene

**Figure 6:**
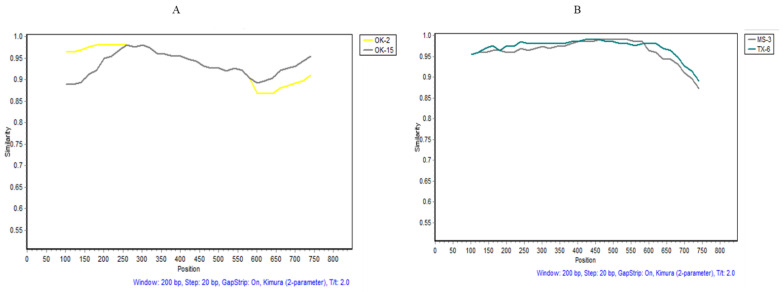
Recombination events analysis confirmed by SimPlot (version 3.5) with a sliding window of 200 bases and a step of 20 bases. Recombination breakpoints between the U.S. watermelon mosaic virus isolates are shown: (**A**) recombination event number 3 (Table 3) in subgroup EM1 isolates (OK15 and OK12), (**B**) recombination event number 3 (Table 3) in subgroup EM5 isolates (MS-3 and TX-6). The *X*-axis shows the nucleotide positions of the coat protein gene while the *Y*-axis shows the percent similarity between the major and minor parent isolates.

The recombination events were examined in the 57 WMV isolates differentiated into three subgroups. Various recombination events (Table 4) were observed in subgroups EM1 and EM5 WMV isolates, but none in EM2 isolates, by different algorithms employed in RDP4 (Table 4). Recombination events that were detected by four or more algorithms [27,28] were considered to be real, while the one detected by two or three algorithms was ignored and removed from the analysis (Table 4). In subgroup EM1 isolates, recombinant event #3 was statistically significant in OK-15 isolate as confirmed by Simplot software (Figure 6A). The potential breakpoints of the recombination event were located at 298–810 nt. The major parent was the OK-2 isolate, while the minor parent was unknown (possible AL-1 isolate) (Table 4). Similarly, in subgroup EM5, recombination event #3 (Table 4) was observed in the MS-3 isolate and the potential breakpoints of the recombination event were located at ~155–724 nt (Figure 6B). The major parent was TX-6 isolates and the minor parent was unknown (possible AL-1) (Table 4).

## 3. Discussion

We have shown for the first time the genetic diversity of 57 WMV isolates from nine southern states of the U.S. that were collected from commercial cucurbit fields. Our study showed that all the 57 U.S. WMV isolates belong to the G3 isolates and none to G1 or G2 isolates. However, within G3, the U.S. isolates divided into three subgroups. The two subgroups (EM1 and EM2) were reported previously in other countries [9,21,22]; none were related to EM3 and EM4, while EM5 (named in this study) was a new distinct subgroup within the G3 that was never reported before within WMV isolates from other countries. 

In 1990, only 1 isolate (Accession no. D13913) reported from Florida [25] was classified as G2 [21], but none of the 57 isolates, including 4 from Florida, belong to G1 or G2 and all of them belong to G3. It is evident that the WMV isolates in the U.S. predominantly belong to G3 isolates and are constantly emerging and expanding the genetic variability, particularly with the existence of the new distinct subgroup EM5 and its isolates. This shows that classical isolates are replaced by G3, which are emerging isolates (EM). Similar observations were also reported in France where EM isolates have replaced the classical WMV isolates [20,22,29]. However, the identification of this new subgroups EM5 in G3 suggest that selection and frequent recombination are the main driving forces for the evolution of G3 isolates which has now possible five subgroups. It is important to note that the new subgroup EM5 isolates have specific aa of G2 isolates, while OK11 and OK 12 isolates have specific aa of both G1 and G2 isolates (Figure 2), which confirms that the above forces are responsible for the emergence of new isolates in G3. Another possible reason could be the wide host range of WMV and its transmission by more than 23 vectors that contribute to the selection and genetic bottleneck of G3 isolates of WMV. Genetic bottlenecks, selection, and founder effects were determined during the horizontal transmission of cucumber mosaic virus (CMV) by two different aphid vectors [30]. 

RNA viruses could accumulate heterogeneous population (termed as quasispecies) in the host due to its error-prone mechanism [31] that provides a pool of genetic variants to be picked by aphid’s vectors during horizontal transmission of the virus. The resulting infection produces a founding population which further increase the pool of genetic variants and could be easily adopted to a particular host. Based on this assumption, the 57 isolates in this study have been collected from various fields and hosts in 9 different states and probably upon introduction in various locations by aphid vectors constituted a founding population that provides a conducive environment for the emergence of new isolates of WMV.

The 57 WMV isolates were collected from various cucurbit hosts, including cucumber, cantaloupe, pumpkin, squash, watermelon, and pigweed (Table 1) in nine different major cucurbit growing states, but interestingly, none of the isolates clustered according to the location or hosts where they were originally isolated, except FL isolates, which clustered together (Figure 1) irrespective of the hosts (cucumber, squash, pumpkin, and watermelon) (Table 1) where they were collected. The possible reason is that the diversity among the FL isolates was very low (<0.5% nt and 0.8% aa) compared with other WMV isolates from other states. However, only four WMV isolates from Florida were analyzed, and meaningful conclusions could be obtained once a large number of isolates from different hosts have been analyzed. Nevertheless, the variability among the U.S. WMV isolates may be due to their frequent introduction from one location to another location through the reservoir hosts, or WMV-infected material during the season or off-season. Similar observations were also noticed previously [24] when 56 WMV isolates from China did not cluster with their geographical locations. It is also possible that WMV is transmitted in the fields located in different states by various vectors and differences in selection, or genetic bottlenecks could be responsible for the evolution of these newly emerged variants, such as subgroup EM5 identified in this work. 

Our study showed that the U.S. WMV isolates have a high degree of variation, up to 10.8% at nt level and 8.5% at aa. In comparison, this variation was higher among the WMV isolates reported from other countries including China (6.6% nt and 3.9% aa) [24] or Slovakian and Iranian WMV isolates (5% nt) [23]. This shows that U.S. WMV isolates are more diverse than those reported in other countries. Natural selection and genetic bottlenecks are the two evolutionary forces that affect the population of plant viruses in nature. In this study, negative selection was detected in the CP gene of the U.S. WMV isolates, which could remove the deleterious mutations and stabilize the genetic structure of the virus populations. The majority of the codons in the CP gene were under negative selection but some were also under positive selection, as detected by REL in the Data monkey software. Positive selection in the CP gene of WMV has also been reported in a recent study [32]. These results show that positive selection is also occurring on individual codons, but to a lower extent compared with negative selection. However, some of the codons in the CP genes that differentiate the three groups of WMV (KET, KEA, or KEKET) or in other genes may play an important role in the virus life cycle, such as encapsidation of the virion; interactions with the RNA genomes of the virus and host specificity for infection may not be under the selection pressure because the virus cannot afford any mutations in such conserved regions to be able to survive.

Gene flow and genetic differentiation analysis among WMV isolates from different states indicated that WMV isolates from Florida were unique and has infrequent gene flow with isolates from other states and were well supported statistically with significant *p* value ((Table 3). This could be due to the long distance between Florida and other states. In contrast, WMV isolates from other states which are in close proximity showed frequent gene flow (Table 3), indicating no significant differences. This notion was well supported by phylogenetic analysis (Figure 1), where WMV isolates from Florida clustered together while WMV isolates from other states did not cluster based on their geographical locations, but instead clustered together in various subgroups including the newly EM5 cluster in G3 (Figure 1). 

While comparing the phylogenetic trees based on CP gene nucleotide sequences (Figure 1) and complete genome sequences (Figure 4), it was noted that few isolates available in both analyses clustered mostly in their respective three major groups (G1, G2, and G3) with some exceptions. For example, the Chinese isolate (Accession no. KF274031) did not cluster in the three major groups (Figure 1 and Figure 3). The complete genome sequences of most of the isolates used in the CP gene analysis were not available in the GenBank database. More than 50% of the 37 isolates used in the complete genome analysis were from France while the remaining isolates came from the rest of the countries. To have more comprehensive phylogenetic analysis, future study shall focus to acquire large number of WMV isolates from other countries and shall be sequenced completely in order to get a detailed comparison of the WMV isolates based on both the CP as well as complete genome sequences. 

Recombination is one of the driving forces of plant virus evolution [33] and has been commonly detected in *Potyviruses* [34,35]. Recombination plays an important role in the evolution of WMV and has been reported previously in WMV populations from other countries [9,20,22] In our analysis, strong recombination events were detected among the U.S. WMV isolates that confirms that genetic recombination is very important in the evolution of global WMV populations. It is also noteworthy to mention that OK-11 isolate was collected from an Amaranthus palmeri (Palmer amaranth) and was a possible recombinant (Table 3) where three algorithms had detected the recombination event in the OK11 WMV isolate. It is clear that WMV isolates not only move through their natural cucurbit hosts but also through various weeds as an infected material.

In conclusion, we showed that the population structure of WMV isolates in the U.S. is highly variable and predominantly belongs to G3 isolates. However, a new subgroup has been identified which includes isolates from Mississippi, Oklahoma, and Texas that is driven by different evolutionary forces, including strong purifying selection and frequent recombination events in nature. We hypothesized that WMV moves from one state to another state through virus-infected material, mostly by human interference. The current information obtained in this study has considerably expanded our knowledge about WMV variability in the U.S. and provides a comprehensive analysis of the population structure of WMV, which could be beneficial in epidemiological studies, as well as in the management of WMV in the U.S. In future, more isolates shall be sequenced from various southern states, including other states where cucurbits are grown, to completely analyze the diversity of WMV and the expansion of the newly detected distinct subgroup (EM5) isolates as well as biological characterization.

## 4. Materials and Methods

### 4.1. Sources of WMV Isolates

During our previous study [11], more than 700 cucurbit leaf samples were collected from 10 different southern states and were tested by dot-immunobinding assay (DIBA) against 17 viruses, including WMV. A total of 57 DIBA-positive WMV samples (Table 1) were randomly selected, representing nine southern states, that included 22 WMV isolates from Texas (TX), 15 from Oklahoma (OK), 7 from Arkansas (AR), 4 each from Florida (FL) and Mississippi (MS), 2 from Georgia (GA), and 1 each from Alabama (AL), Kentucky (KY), and Louisiana (LA). 

### 4.2. RNA Extraction, Amplification, and Sequencing

Total RNA was extracted from the DIBA-positive samples as reported before [35]. Two-step reverse transcription-polymerase chain reaction (RT-PCR) was performed to amplify the CP gene (849 nt) of WMV using WMV specific primers: forward 5′-AACACACAACCAAGT-3′ and reverse 5′-TAACGACCCGAAATGCTAACT-3′, as described previously [36]. The PCR products were analyzed on a 1% agarose gel, and purified using a QIAquick PCR Purification Kit (QIAGEN, Germantown, MD, USA). Purified PCR products were cloned using pGEM®-T vector (Promega, Madison, WI, USA), and transformed into Escherichia coli DH5α competent cells (New England Biolabs, Ipswish, MA, USA). Three to five positive recombinant clones were sequenced in both directions using dye terminator cycle sequencing (Applied Biosystems 3130 genetic analyzer) at the core facility lab, Department of Biological Science, the University of Tulsa, Oklahoma.

### 4.3. Consensus Sequences and Phylogenetic Analysis of the CP Gene

Sequences of the complete CP gene of all 57 WMV isolates were submitted to GenBank with the accession numbers from MG021247 to MG021303 (Table 1). For comparison, complete CP sequences of 89 WMV isolates, reported from 13 different countries and including representative isolates of all the three reported groups (Group 1, 2, and 3), were obtained from GenBank database (Table S1) and used in phylogenetic analysis.

Consensus sequences were obtained from the alignment of three-five clones for each individual WMV isolate using EditSeq ™ and MegAlign ™ within the DNASTAR suite of programs (Madison, WI, USA). Multiple sequence alignment was performed using the Clustal X program [37] and BioEdit [38]. A phylogenetic tree was constructed using maximum likelihood (ML) in Mega 7 with 1000 bootstrap replications [38]. The Tamura-Nei model (TN93+G+I) [39,40] was used for making a ML tree of the complete CP sequences as a result of model tests in Mega7 for determining the best model of nucleotide substitution. The CP sequence of soybean mosaic virus (SMV) was used as an outgroup. Trees were visualized in Figtree v.1.3.1 [41].

### 4.4. Selection Pressure Analysis

Selection pressure on the CP gene was determined using SNAP [42], as well as for each codon using the Datamonkey online positive selection interface [43]. The ratio of dN/dS (ω) was estimated for the CP gene, where dN means the average number of non-synonymous substitutions per non-synonymous site and dS represents the average number of synonymous substitutions per synonymous site [44]. Selection pressure was considered negative, or purifying, when the ratio of ω < 1, or positive, or diversifying, when ω > 1, and neutral when ω = 1. To detect positive selection on each codon, three different methods were used: single likelihood ancestor counting (SLAC), random effects likelihood (REL), and fixed effects likelihood (FEL), with default parameters including the significance level [43]. The HKY85 nucleotide substitution biased model available on the Datamonkey server was used for all analyses (SLAC, FEL and REL) by selecting the automatic model selection [43]. To measure the synonymous codon usage biases in the CP gene, the effective number of codons (ENC) was calculated using DnaSPv6 [45]. ENC values range from 20 to 61 for a gene [46], where the value of 20 indicates that one codon is used for each amino acid (extreme biased), while a value of 61 shows that all codons are used equally (no bias). 

### 4.5. Genetic Differentiating and Gene Flow Analysis

Genetic differentiation, gen flow among, and permutation-based statistical tests Ks, Ks*, Z*, and Snn among WMV isolates collected from different states were measured in DnaSP6v6 using F statistics (FST) [45]

### 4.6. Co-Evolution Analysis of CP Amino Acids

Evidence of coevolution sites in the CP gene sequences of WMV isolates was evaluated using the Bayesian Graphical Models (BGM) method implemented in Spidermoneky through the Datamonkey web-based interface [47].

### 4.7. Phylogenetic Analysis of the Complete Genome Sequences

In order to confirm the WMV grouping (G1, G2, and G3) based on the CP gene sequence, complete genome sequences of selected 37 WMV isolates including 2 U.S. isolates and 35 from 12 different countries representing all the three groups (G1, G2, and G3) were downloaded from GenBank NCBI database and used in the phylogenetic analysis (Table S3). One of the U.S. WMV isolates (Accession no. HQ384216) collected from *Dendrobium anosmum* (orchid) and directly submitted to GenBank while the other U.S. WMV isolate (Accession no. KU246036) was collected from watermelon in Texas during our previous study [11]. Later, it was sequenced in our lab [48] and used for comparison in the complete genome analysis of WMV isolates. 

A maximum likelihood (ML) tree with 1000 bootstrap replications [39] was constructed based on the best-fit model of nucleotide substitution in Mega 7. The general time-reversible substitution model with a gamma distribution and invariant sites (GTR+G+I) was used for making a ML tree of the complete genome sequences while soybean mosaic virus (SMV) (Accession number AJ628750) was used as an outgroup. Trees were visualized in Figtree v.1.3.1 [41]. 

The pairwise nucleotide sequence identities scores among the 37 WMV isolates were determined using SDT software version 1.2 [49].

### 4.8. Detection of Recombination Events

The recombination events were investigated in the complete CP gene nucleotide sequences as well as complete genome sequences using RDR4 Beta 4.69, implementing all seven different recombination detection algorithms with default parameters [50]. Only potential recombination events supported by at least four RDR4-implemnted algorithms coupled with phylogenetic evidence of recombination were included in the analysis of recombination events. Furthermore, the potential recombination was also confirmed by Simplot software version 3.5 [51] using a window of 200 nt and step size of 20 nt.

## Figures and Tables

**Table 1:** *Watermelon mosaic virus* isolates collected in nine different states of the U.S. and analyzed in this study.

No	Name of State	Name of Isolate	Host	Year Collected	Accession Number
1	Alabama	AL-1	Watermelon	2010	MG021270
2	Arkansas	AR-1	Pumpkin	2010	MG021275
3		AR-2	Watermelon	2010	MG021276
4		AR-3	Pumpkin	2010	MG021277
5		AR-4	Cantaloupe	2010	MG021278
6		AR-5	Watermelon	2010	MG021279
7		AR-6	Watermelon	2010	MG021280
8		AR-7	Watermelon	2010	MG021281
9	Florida	FL-1	Squash	2011	MG021262
10		FL-2	Pumpkin	2011	MG021263
11		FL-3	Cucumber	2011	MG021264
12		FL-4	Watermelon	2011	MG021265
13	Georgia	GA-1	Watermelon	2010	MG021271
14		GA-2	Watermelon	2010	MG021272
15	Kentucky	KY-1	Watermelon	2010	MG021273
16	Louisiana	LA-1	Watermelon	2010	MG021274
17	Mississippi	MS-1	Cantaloupe	2007	MG021266
18		MS-2	Squash	2007	MG021267
19		MS-3	Squash	2008	MG021268
20		MS-4	Watermelon	2010	MG021269
21	Oklahoma	OK-1	Squash	2008	MG021247
22		OK-2	Watermelon	2010	MG021248
23		OK-3	Watermelon	2009	MG021249
24		OK-4	Watermelon	2009	MG021250
25		OK-5	Watermelon	2009	MG021251
26		OK-6	Cantaloupe	2009	MG021252
27		OK-7	Watermelon	2010	MG021253
28		OK-8	Watermelon	2010	MG021254
29		OK-9	Watermelon	2010	MG021255
30		OK-10	Watermelon	2010	MG021256
31		OK-11	Palmer amaranth	2010	MG021257
32		OK-12	Pumpkin	2010	MG021258
33		OK-13	Watermelon	2010	MG021259
34		OK-14	Watermelon	2010	MG021260
35		OK-15	Pumpkin	2010	MG021261
36	Texas	TX-1	Watermelon	2010	MG021282
37		TX-2	Cucumber	2010	MG021283
38		TX-3	Watermelon	2010	MG021284
39		TX-4	Watermelon	2010	MG021285
40		TX-5	Watermelon	2010	MG021286
41		TX-6	Watermelon	2010	MG021287
42		TX-7	Watermelon	2010	MG021288
43		TX-8	Watermelon	2010	MG021289
44		TX-9	Watermelon	2010	MG021290
45		TX-10	Watermelon	2010	MG021291
46		TX-11	Watermelon	2010	MG021292
47		TX-12	Watermelon	2010	MG021293
48		TX-13	Watermelon	2010	MG021294
49		TX-14	Watermelon	2010	MG021295
50		TX-15	Watermelon	2010	MG021296
51		TX-16	Watermelon	2010	MG021297
52		TX-17	Watermelon	2010	MG021298
53		TX-18	Watermelon	2010	MG021299
54		TX-19	Watermelon	2010	MG0212300
55		TX-20	Watermelon	2010	MG0212301
56		TX-21	Watermelon	2010	MG0212302
57		TX-22	Watermelon	2010	MG0212303

**Table 2:** Selection pressure acting on the coat protein (CP) gene of watermelon mosaic virus isolates from the U.S.

Subgroups within G3 (EM Isolates)	No. of Isolates ^a^	ENC ^b^	No. of Nnegatively ^c^ Selected Codons			*dNS* ^d^	Variance (*d_NS_*)	Standard Deviation (*d_NS_*)	*dS* ^e^	Variance (*d_NS_*)	Standard Deviation (*d_NS_*)	*dNS/dS*
			SLAC	FEL	REL							
EM1	17	50.092	2	29	55	0.0129	0.0000	0.0022	0.0639	0.0000	0.0098	0.2019
EM2	31	51.828	9	25	1	0.0148	0.0000	0.0024	0.0479	0.0001	0.0077	0.3089
EM5	9	50.573	2	17	31	0.0188	0.0001	0.0037	0.0906	0.0002	0.0154	0.2075

^a^ Number of WMV isolates in each subgroup within G3 (emerging isolates). EM-V is the new distinct subgroup identified in this work. ^b^ ENC- effective number of codons, were estimated by DnaSPv6 (see materials and methods). ^c^ Number of negatively selected codons was calculated suing SLAC, FEL, and REL programs within Datamonkey software ^d^ Average number of non-synonymous substitutions per non-synonymous site. ^e^ Average number of synonymous substitutions per synonymous site. ^d,e^ *d_NS_* and *d_S_* along with standard variation and variance was calculated using SNAP (non-synonymous analysis program) (see materials and methods).

**Table 3:** Estimates of genetic differentiation and gene flow among different population of watermelon mosaic virus isolates from different states.

States	Fst	Nm	Ks	Kst	*p*-Value	Ks*	Kst*	*p*-Value	Z*	*p*-Value	Snn	*p*-Value
AR vs. FL	0.439	0.32	30.54	0.246	0.008 **	3.06	0.112	0.007 **	2.66	0.006 **	0.95	0.008 **
AR vs. MS	0.083	2.73	43.03	0.043	0.180 ns	3.54	0.021	0.095 ns	2.88	0.051 ns	0.95	0.007 **
AR vs. OK	0.004	65.87	48.56	0.001	0.364 ns	3.68	0.000	0.036 ns	4.44	0.293 ns	0.772	0.013 *
AR vs. TX	0.061	3.84	44.73	0.024	0.100 ns	3.52	0.013	0.082 ns	4.94	0.051 ns	0.84	0.004 **
AR vs. OS ^a^	0.019	12.93	46.84	0.010	0.025 ns	3.66	-0.000	0.280 ns	3.19	0.319 ns	0.83	0.028 *
Fl vs. MS	0.620	0.15	22.0	0.483	0.024 *	2.42	0.279	0.024 *	8.62	0.024 *	1.00	0.024 *
FL vs. OK	0.470	0.28	40.58	0.174	0.001 **	3.40	0.079	0.0010 **	3.86	0.0010 **	1.00	0.002 **
FL vs. TX	0.569	0.19	38.45	0.186	0.001 **	3.31	0.076	0.000 ***	4.52	0.0010 **	1.00	0.000 ***
FL vs. OS	0.270	0.68	29.42	0.158	0.028 *	2.83	0.120	0.036 *	2.29	0.028 *	0.888	0.024 *
MS vs. OK	0.010	24.29	47.80	0.003	0.035 ns	3.63	0.009	0.192 ns	4.08	0.115 ns	0.921	0.005 **
MS vs. TX	0.026	9.07	43.73	0.007	0.307 ns	3.46	0.012	0.125 ns	4.69	0.040 *	0.961	0.003 **
MS vs. OS	0.128	1.69	44.68	0.074	0.130 ns	3.51	0.033	0.157 ns	2.48	0.112 ns	0.833	0.044 *
OK vs. TX	0.027	8.88	46.81	0.014	0.162 ns	3.56	0.019	0.022 *	5.39	0.005 **	0.887	0.000 ***
OK vs. OS	0.024	10.06	49.86	0.009	0.315 ns	3.68	0.006	0.278 ns	4.21	0.181 ns	0.850	0.014 *
TX vs. OS	0.122	1.79	45.41	0.043	0.097 ns	3.51	0.024	0.041 *	4.75	0.020 *	0.944	0.000 ***

*p* value estimates are based on probability obtained by the permutation test with 1000 replicates. ns, not significant; *, 0.01 < *p* < 0.05; **, 0.001 < *p* < 0.01; ***, *p* < 0.001. *p* > 0.001 significantly rejects the null hypothesis that there is no genetic differentiation between two populations. ^a^ WMV isolates from other states include: two isolates from Georgia and one isolate each from Alabama, Louisiana, and Kentucky.

**Table 4:** Recombination events in the nucleotide sequence of the coat protein (CP) gene of watermelon mosaic virus isolates from the U.S.

Sub-Groups ^a^	Event No.	Recomb.	Major Parent	Minor Parent	Recombination Sites			Detection Methods				
EM1 Isolates						RDP	GENECON V	Bootscan	Maxchi	Chimaera	Sisscan	3-Seq
	1	AR-3	TX-13	Unknown (Al-1)	69–735 nt	-	-	-	8.115 × 10^−2^	-	-	2.864 × 10^−2^
	2	OK-2	TX-13	Unknown (Al-1)	680–832 nt	-	-	-	8.115 × 10^−2^	-	-	2.864 × 10^−2^
	3 ^b^	OK-15	OK-2	Unknown(Al-1)	298–810 nt	1.243 × 10^−4^	4.828 × 10^−3^	-	6.683 × 10^−8^	2.881 × 10^−5^	-	9.765 × 10^−9^
EM2	None											
EM5 isolates												
	1	OK-11	MS-3	OK-12	230–824 nt				8.231 × 10^−4^		4.035 × 10^−10^	1.655 × 10^−6^
	2	TX-7	TX-5	OK-12	627–743 nt		3.198 × 10^−2^					1.201 × 10^−4^
	3 ^b^	MS-3	TX-6	Unknown (OK-12)	155–724 nt				7.921 × 10^−3^	1.214 × 10^−1^	4.096 × 10^−2^	4.814 × 10^−3^
	4	OK-13	OK-12	TX-6	58–783 nt				3.987 × 10^−3^	6.198 × 10^−3^		

^a^ All three subgroups are within G3 (Emerging isolates) of WMV; ^b^ recombination event detected by at least four algorithms was considered statistically significant.

## Data Availability

All 57 coat protein gene sequences of WMV isolates from the US presented in this study (Table 1) were submitted to NCBI database. The accession numbers from (MG021270- MG0212303) can be found online https://www.ncbi.nlm.nih.gov/genbank/ (accessed on 30 August 2021).

## Supplementary Materials

The following are available online at https://www.mdpi.com/article/10.3390/pathogens10101245/s1, Table S1: Nucleotide sequences of the coat protein gene of watermelon mosaic virus isolates downloaded from GenBank [9,24,25,52,53,54,55,56,57]. Table S2: Selection pressures in the coat protein (CP) gene of watermelon mosaic virus isolates from the U.S. Table S3: Complete genome sequences of watermelon mosaic virus isolates downloaded from GenBank [10,48,52,53,57,58]. Table S4: Potentially co-evolving sites in CP gene sequences of watermelon mosaic virus isolates identified using Bayesian Graphical Models (BGM).
