# Supplementary material for: Genetic Variability and Evidence of a New Subgroup in Watermelon Mosaic Virus Isolates
Source: Pathogens. 2021 Sep 26;10(10):1245. doi: 10.3390/pathogens10101245 (PMC8538135; doi:10.3390/pathogens10101245)
Supplement: Supplementary file 1 [file pathogens-10-01245-s001.zip › pathogens-1359755-supplementary.pdf]

pathogens

MDPI

Table S1 Nucleotide sequences of the coat protein gene of *watermelon mosaic virus* isolates downloaded from GenBank.

No.	Accession	Country	Molecular Group	Year	References
1	KP164988	Argentina	G3	2012	Unpublished
2	EU660580	Chile	G2	1987	[52]
3	EU660582		G1	2002	[52]
4	EF127832	China	G3	-	Unpublished
5	AY464948		G2	-	Unpublished
6	EF122501		G3	2006	Direct submission
7	DQ399708		G2	2006	Direct submission
8	JX079685		G3	2014	Direct submission
9	KF274031		G1	2014	Direct submission
10	KM527438		G3	2013	[24]
11	KM527439		G3	2013	[24]
12	KM527440		G3	2013	[24]
13	KM527441		G3	2013	[24]
14	KM527442		G3	2013	[24]
15	KM527443		G3	2013	[24]
16	KM527444		G3	2013	[24]
17	KM527445		G3	2014	[24]
18	KM527446		G3	2014	[24]
19	KM527447		G3	2014	[24]
20	KM527448		G3	2014	[24]
21	KM527449		G3	2014	[24]
22	KM527450		G3	2014	[24]
23	KM527451		G3	2014	[24]
24	KM527452		G3	2014	[24]
25	KM527453		G3	2014	[24]
26	KM527454		G3	2013	[24]
27	KM527455		G3	2013	[24]
28	KM527456		G3	2013	[24]
29	KM527457		G3	2013	[24]
30	KM527458		G3	2013	[24]
31	KM527459		G3	2013	[24]
32	KM527460		G3	2013	[24]
33	KM527461		G3	2013	[24]
34	KM527462		G3	2013	[24]

35	KM527463		G3	2013	[24]
36	KM527464		G3	2013	[24]
37	KM527465		G3	2013	[24]
38	KM527466		G3	2013	[24]
39	KM527467		G3	2014	[24]
40	KM527468		G3	2014	[24]
41	KM527469		G3	2014	[24]
42	KM527470		G3	2014	[24]
43	KM527471		G3	2014	[24]
44	KM527472		G3	2014	[24]
45	KM527473		G3	2013	[24]
46	KM527474		G3	2013	[24]
47	KM527475		G3	2014	[24]
48	KM527476		G3	2014	[24]
49	KM527477		G3	2014	[24]
50	KM527478		G3	2014	[24]
51	KM527479		G3	2014	[24]
52	KM527480		G3	2013	[24]
53	KM527481		G3	2013	[24]
54	KM527482		G3	2013	[24]
55	KM527483		G3	2013	[24]
56	KM527484		G3	2013	[24]
57	KM527485		G3	2013	[24]
58	KM527486		G3	2014	[24]
59	KM527487		G3	2014	[24]
60	KM527488		G3	2014	[24]
61	KM527489		G3	2014	[24]
62	KM527490		G3	2014	[24]
63	KM527491		G3	2013	[24]
64	KM527492		G3	2013	[24]
65	KM527493		G3	2013	[24]
66	AY437609	France	G1	1972	[9]
67	JF273464		G3	2007	[53]
68	EU660583		G3	2003	[52]
69	EU660585		G3	2005	[52]
70	EU660578		G1	2000	[52]
71	EU660584	Iran	G1	2002	[52]

72	AF322376	Israel	G2	2000	Unpublished
73	EU660590	Italy	G1	2000	[52]
74	AB693979	Japan	G3	1996	Unpublished
75	AB001994		G3	1997	Unpublished
76	AJ579483	Spain	G1	1999	[54]
77	AJ579484		G1	1999	[54]
78	AJ579524		G1	1999	[54]
79	AJ579523		G1	1999	[54]
80	AJ579522		G1	1999	[54]
81	AJ579481		G2	1999	[54]
82	KF637299	South Korea	G1	2014	Unpublished
83	L229071	Tonga	G2	1993	[55]
84	EU660579	Turkey	G1	1991	[52]
85	JX028595	USA	G3	2010	[56]
86	JX028594		G3	2010	[56]
87	D13913		G2	1990	[25]
88	KC292915	Venezuela	G2	2010	[57]
89	AJ628750	SMV	outgroup	2004	

Table S2 Selection pressures in the coat protein (CP) gene of *watermelon mosaic virus* isolates from the U.S.

No of isolates	States	d_{NS}	Variance (d_{NS})	Standard deviation (d_{NS})	d_S	Variance (d_{NS})	Standard deviation (d_{NS})	d_{NS}/d_S
7	Arkansas	0.0194	0.0000	0.0052	0.2256	0.0011	0.0331	0.0859
4	Florida	0.0039	0.0000	0.0021	0.0123	0.0000	0.0062	0.3170
4	Mississippi	0.0179	0.0000	0.0064	0.1911	0.0008	0.0297	0.0937
15	Oklahoma	0.0229	0.0000	0.0052	0.2434	0.0008	0.0294	0.0940
22	Texas	0.0189	0.0000	0.0048	0.2284	0.0007	0.0277	0.0827
5	Others states	0.0208	0.0000	0.0059	0.2454	0.0013	0.0354	0.0847

[a] WMV isolates from other states include: two isolates Georgia and one isolate each from Alabama, Louisiana and Kentucky.

Table. S3 Complete genome sequences of *watermelon mosaic virus* isolates downloaded from GenBank

No.	Accession	Isolate name	Country	Year	Genome (nt)	References
1	KP164988	WMV 1 SDE FF	Argentina	2012	10027	Direct submission
2	EU660580	CHI87-620	Chile	1987	10045	[52]
3	EU660582	CHI02-481	Chile	2002	10041	[52]
4	DQ399708	WMV-CHN	China	2006	10037	Direct submission
5	JXO79685	ShanXi	China	2012	10046	Direct submission
6	KF274031	None	China	2013	10063	Direct submission
7	JF273469	C04-106	France	2004	10047	[53]
8	JF273467	Cg09-640	France	2009	10032	[53]
9	NC_006262	WMV-Fr	France	2003	10035	[53]
10	JF273466	A08-170	France	2008	10031	[53]
11	JF273465	A08-160	France	2008	10031	[53]
12	JF273464	C07-014	France	2007	10031	[53]
13	JF273463	C06-257	France	2006	10049	[53]
14	JF273462	C06-526	France	2006	10036	[53]
15	JF273461	C07-349	France	2007	10040	[53]
16	JF273460	C05-465	France	2005	10046	[53]
17	JF273459	C05-464	France	2005	10038	[53]
18	JF273458	C05-463	France	2005	10041	[53]
19	EU660589	C05-337	France	2005	10041	[52]
20	EU660588	C06-666	France	2006	10045	[52]
21	EU660587	C06-188	France	2006	10042	[52]
22	EU660586	FBR04-37	France	2004	10051	[52]
23	EU660585	C05-270	France	2005	10051	[52]
24	EU660583	FMF03-141	France	2003	10050	[52]
25	EU660581	FMF00-LL1	France	2000	10036	[52]
26	EU660578	FMF00-LL2	France	2000	10040	[52]
27	KM597071	RKG2	India	2014	10030	Direct submission
28	KM597070	RKG	India	2014	10030	Direct submission
29	EU660584	IR02-54	Iran	2002	10045	[52]
30	EU660590	ITA00-G	Italy	2000	10041	[52]
31	FJ823122	Lecce	Italy	2012	10045	[58]
32	AB218280	WMV-Pk	Pakistan	2005	10039	[10]
33	AB369278	Watermelon	South Ko-rea	2007	10037	Direct submission
34	EU660579	TURK91	Turkey	1991	10042	[52]

35	KC292915	VE10-099	Venezuela	2013	10039	[59]
36	HQ384216	Dendrobium	USA	2010	10057	Direct submission
37	KU246036	TX29	USA	2010	10049	[48]
38	NC002634	Soybean mosaic virus		2011	9588	Direct submission

Table S4. Potentially co-evolving sites in CP gene sequences of *watermelon mosaic virus* isolates identified using Bayesian Graphical Models (BGM).

Site 1[a]	Site 2[a]	P [Site 1 –> Site 2][b]	P [Site 2 –> Site 1][c]	P [Site 1 <–> Site 2][d]	Site 1 subs[e]	Site 2 subs[e]	Shared subs[f]
2	4	0.24	0.27	0.51	1	1	1
7	13	0.33	0.3	0.62	1	1	1
12	5	0.34	0.29	0.63	1	1	1
23	96	0.4	0.38	0.78	4	2	2
26	33	0.22	0.35	0.57	1	1	1
95	222	0.33	0.33	0.66	1	1	1
140	241	0.29	0.29	0.58	1	1	1
171	262	0.27	0.29	0.55	1	1	1
201	247	0.44	0.37	0.81	2	2	2
201	251	0.31	0.22	0.53	2	3	2
234	235	0.52	0.41	0.94	2	2	2
234	236	0.4	0.094	0.5	2	4	2
239	246	0.76	0.22	0.98	3	5	3
248	249	0.36	0.28	0.64	1	1	1
258	259	0.36	0.61	0.96	5	3	3
263	275	0.19	0.52	0.71	4	2	2

[a] - Site 1 and Site 2 are the coevolving sites, with posterior probability >= 0.5

[b] - Probability that Site 2 is conditional dependent on Site 1

[c] - Probability that Site 1 is conditional dependent on Site 2

[d] - Probability that Sites 1 and 2 are conditionally independent

[e] - Number of inferred substitutions at the site

[f] - Number of inferred shared substitutions
